# Supplementary material for: Helheim Glacier ice velocity variability responds to runoff and terminus position change at different timescales
Source: Nat Commun. 2022 Oct 12;13:6022. doi: 10.1038/s41467-022-33292-y (PMC9556534; doi:10.1038/s41467-022-33292-y)
Supplement: Supplementary file 1 — Supplementary information [file 41467_2022_33292_MOESM1_ESM.pdf]

Supplementary Information for "Helheim Glacier ice velocity variability responds to runoff and terminus position change at different timescales"

Lizz Ultee[1,2,3*], Denis Felikson[4,5], Brent Minchew[3], Leigh A. Stearns[6], Bryan Riel[3,7]

[1]Dept. of Earth & Climate Sciences, Middlebury College, Middlebury, VT, USA
[2]School of Earth & Atmospheric Sciences, Georgia Institute of Technology, Atlanta, GA, USA
[3]Dept. of Earth, Atmospheric, and Planetary Sciences, Massachusetts Institute of Technology, Cambridge, MA, USA
[4]Cryospheric Sciences Laboratory, NASA Goddard Space Flight Center, Greenbelt, MD, USA
[5]Goddard Earth Sciences Technology and Research II, Morgan State University, Baltimore, MD, USA
[6]Department of Geology, University of Kansas, Lawrence, KS, USA
[7]School of Earth Sciences, Zhejiang University, Hangzhou 310027, China

Introduction

This supplement includes additional description of statistical methods with accompanying figures, as well as more detailed text and figures to aid interpretation of the main text.

Note S1 Physical and statistical interpretation of cross-correlations

The cross-correlation at lag k, given in main text Equation 2, is a measure of the similarity of two series when one is offset from the other by k time steps. Cross-correlations computed for a range of lag values can be visualized in a correlogram such as Figure S1, with peak locations indicating the time delay for which series f appears to influence series v most strongly.

For some applications, the sign of the lag at maximum cross-correlation is used to interpret whether changes in series f are potentially causative for

*eultee@middlebury.edu

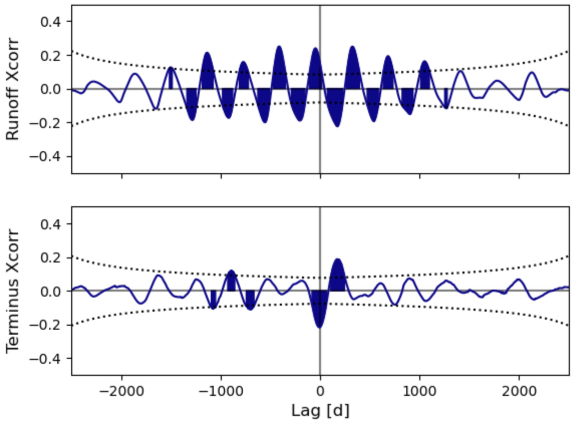

Figure S1: Example correlograms for full, single-differenced signals at the point nearest the terminus. Dashed lines indicate 95% significance limits around 0, scaled for each variable as described in Section 2 and shown in Figure S2. The cross-correlation of runoff and velocity is nearly symmetric; the three maxima at positive and negative lags closest to 0 lag differ by less than 1×10^{-3}. The cross-correlation of velocity with terminus position is less symmetric and shows only two significant extrema close to 0 lag.

changes in series v or vice versa. For our analysis of velocity response to changes in forcing variables, we considered only positive lags. While there may be cross-correlations significantly different from 0 at negative lags, their physical meaning differs per variable. For example, the quasi-periodic nature of surface mass balance and runoff is likely to produce significant cross-correlations at negative lags (Figure S1), even though there is no physical reason to expect feedback from velocity to either variable at seasonal to multi-annual time scales. Terminus position, by contrast, can respond to velocity variability at the time scales we analyse. As such, a strong cross-correlation at negative lag could be interpreted to indicate terminus position responding to changes in velocity. We focus our interpretation on velocity responses to forcing (positive lags) but present both positive and negative lags in this Supplement for transparency.

Note S2 Correction to significance limits for autocorrelated data

We processed our data as described in Section 2.4, single-differencing the series and modifying significance limits, to reduce the risk of spurious cross-correlations. Figure S2 shows the autocorrelations and correction factors F of all variables. Before differencing, each of the series had some autocorrelation (left column of Figure S2). After differencing, the velocity time series had strong autocorrelation, but none of the other single-differenced full series did, and the correction factors F were relatively small (center column of Figure S2). The long-term-varying series all had strong autocorrelation. Therefore

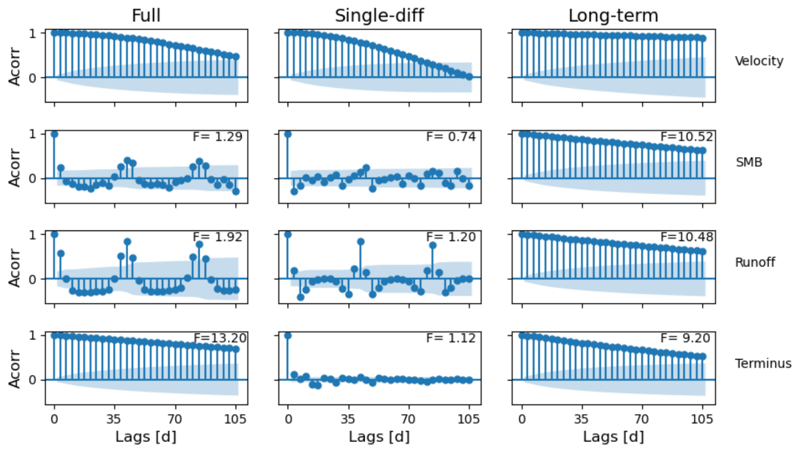

Figure S2: Autocorrelations of each time series analysed, for the full signals (left), single-differenced signals (center), and un-differenced, long-term-varying components (right). Correction factors F for the significance limits on cross-correlation of each variable with velocity are shown in the upper right corner of each panel.

the factor F modifying the significance limit for each set of long-term-varying cross-correlations was large (right column of Figure S2), resulting in our finding of no significant cross-correlations with surface mass balance or runoff in the long-term case.

The quantitative differences among cross-correlations with SMB, runoff, and terminus position are not affected by the factors F. If we had not modified the significance limits, we would have found qualitatively similar results, with the sole difference being that we could have interpreted the long-term-varying runoff and surface mass balance results as significantly different from 0.

Note S3 Phase shift in differenced signals

All cross-correlations that we present in Section 3.1 and 3.2 are computed on single-differenced signals, as described in Section 2. The differencing is necessary to produce statistically meaningful cross-correlations, as Augmented Dickey-Fuller and KPSS tests showed that the original signals were not stationary and the differenced signals were. It is important to note the phase shift that arises from differencing a quasi-periodic signal, which is especially noticeable in the single-year cross-correlations (Figures 3 and S3). For example, Figure S3 shows that in several years including 2010, 2012, 2013, and 2015, there is a peak in positive cross-correlation at short negative lag times. We have plotted the velocity and runoff signals and their differenced versions for 2013 in Figure S4 below. Figure S4 shows that although the peak in runoff does precede the velocity peak in 2013, the peak in differenced velocity is offset backward relative to the peak in differenced runoff. The two signals have different shapes—for example, runoff is more triangular than velocity—and so the phase shift affects

them differently.

Note S4 Choice of interpolation method

We applied the flexible time series method of [1] to the velocity time series and used piecewise linear interpolation via scipy.interpolate.interp1d to sample the other series at equal spacing, as described in Section 2. We also tested 'slinear', 'quadratic', and 'cubic' settings in the scipy interpolation and found practically identical correlograms. Therefore we have presented the results from piecewise linear interpolation for simplicity. Readers wishing to explore other interpolation schemes, for example using the ALPS tool described in [2], are invited to access our GitHub repository 'nifl' (see Acknowledgements), which includes helper functions for analysing cross-correlations generated by an interpolation scheme of choice.

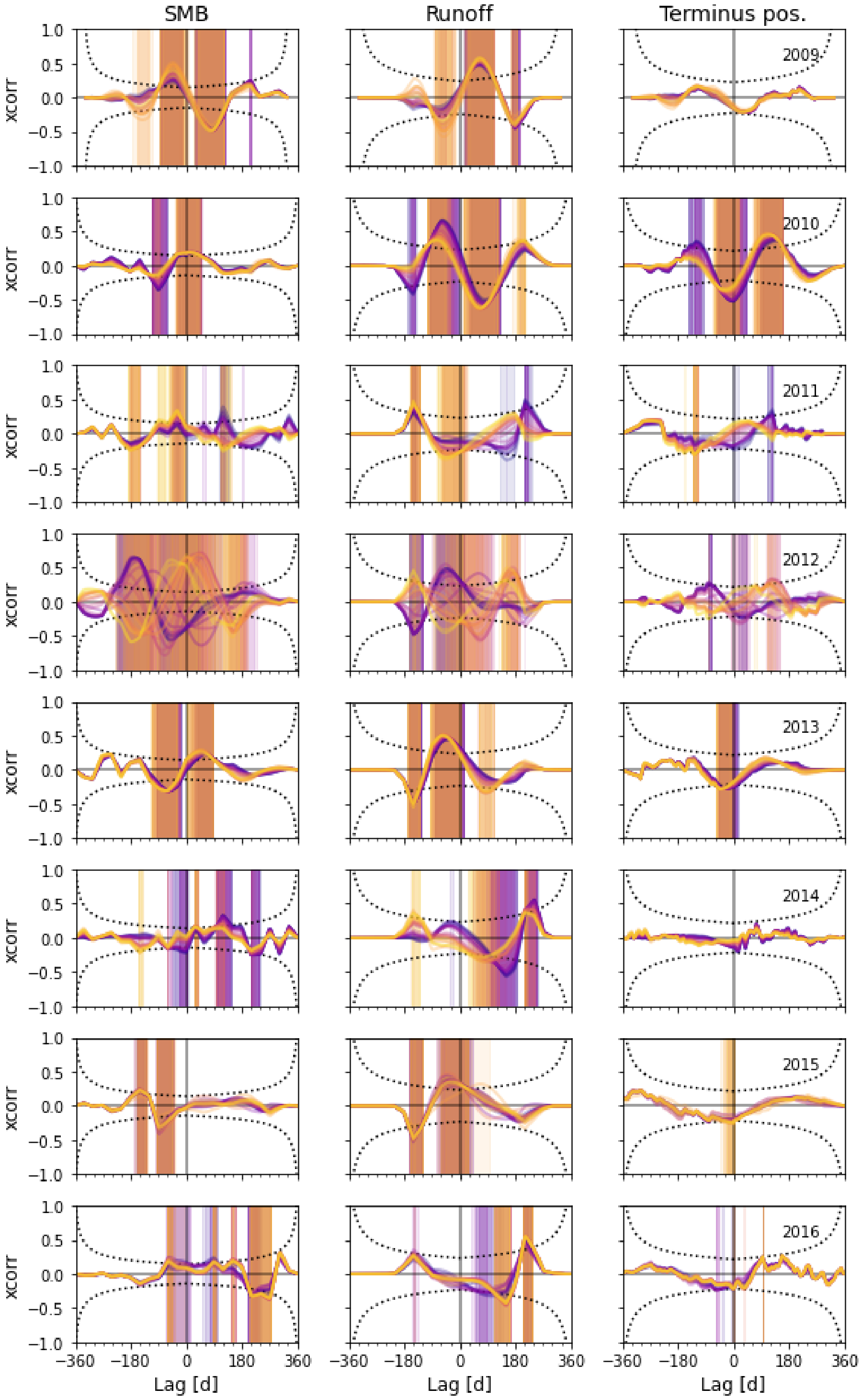

Figure S3: Cross-correlation between annual subsets of surface speed and system variables, as in main text Figure 3, presented here for both positive and negative lag. Curve and fill colors correspond to the position of each point along the flowline as marked in Figure 1A, C, and D; brighter yellow colors are upstream points and dark purple points are downstream.

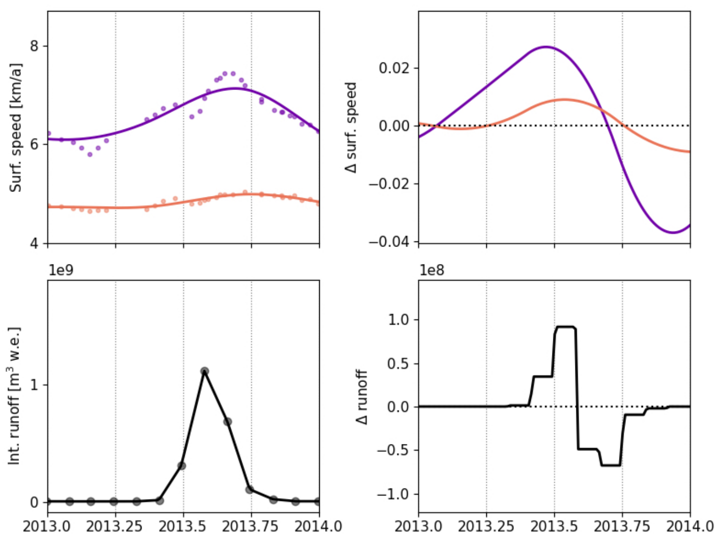

Figure S4: Example velocity and runoff signals (left) and their differenced versions (right). Differencing of a quasi-periodic function produces a phase shift, which is reflected in the annual subset cross-correlations in main text Figure 3 and Figure S3 above.

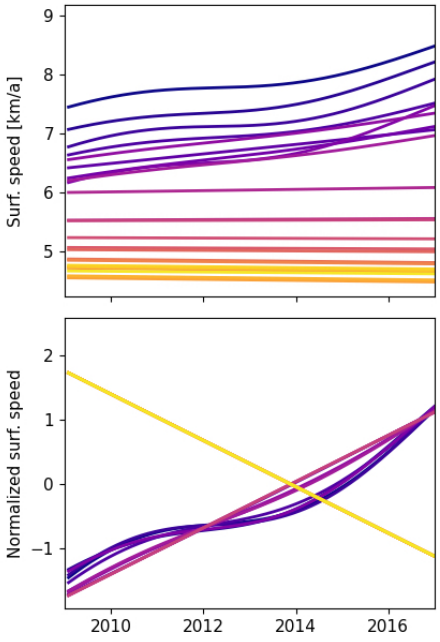

Figure S5: (Top) Long-term-varying velocity for all points along the flowline, shown here with vertical exaggeration to improve readability; and (bottom) normalized signals $\hat{v} = (v - \bar{v})/\sigma(v)$ on which cross-correlation is computed (see Section 2).

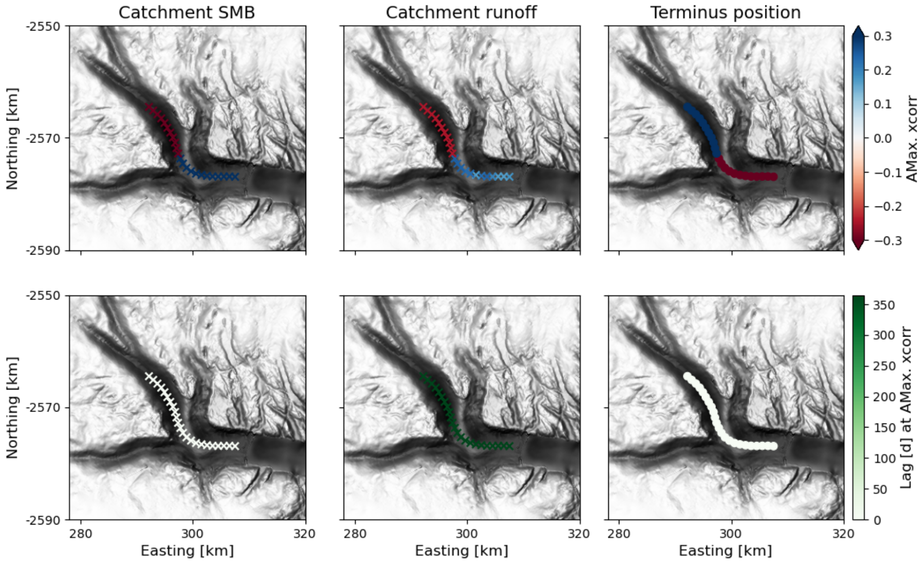

Figure S6: Cross-correlation of largest absolute value (top row) and corresponding lag (bottom row) between the long-term varying components of ice surface speed and each variable (columns). Colorbars for cross-correlation and lag used here are consistent with main text Figure 2 to allow intercomparison; however, the range of values represented here exceeds those shown on that figure. Circles and crosses indicate values that are and are not significant at the 95% confidence level, respectively.

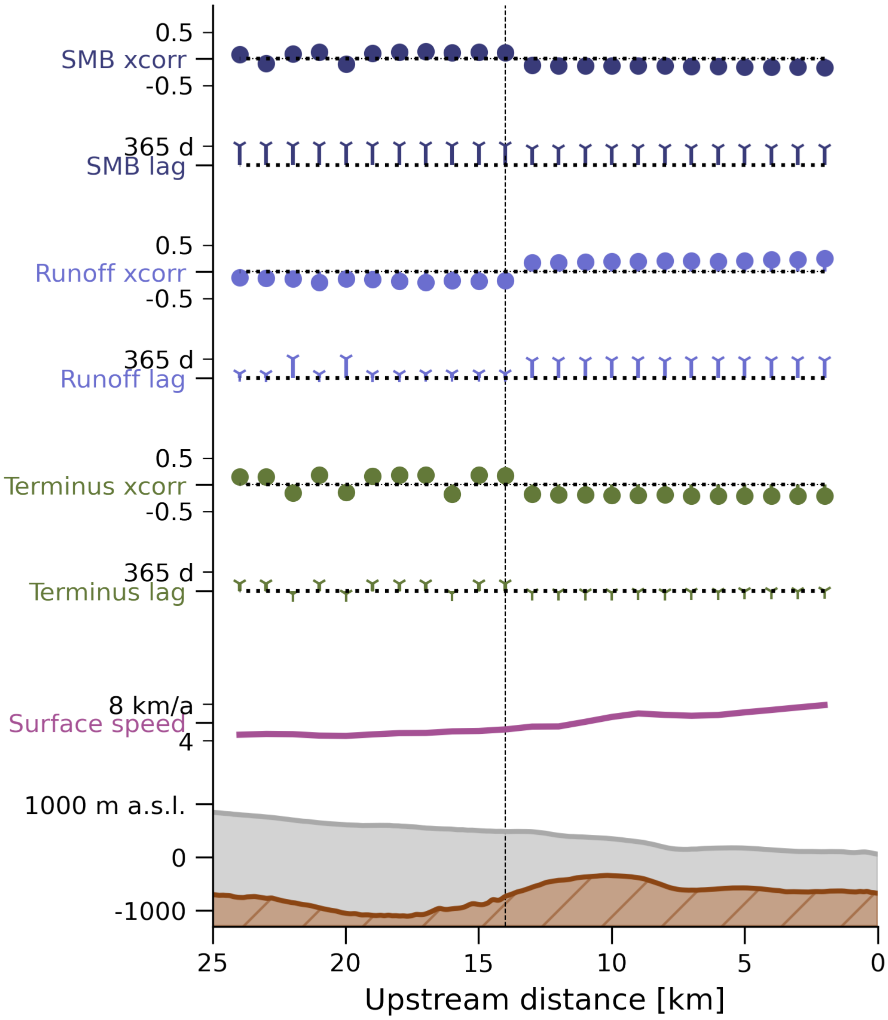

Figure S7: Along-flow view of the short-term cross-correlations and lag times at the absolute maximum cross-correlation for all three variables.

References

[1] B. Riel, B. Minchew, and I. Joughin. Observing traveling waves in glaciers with remote sensing: new flexible time series methods and application to Sermeq Kujalleq (Jakobshavn Isbræ), Greenland. *The Cryosphere*, 15(1):407–429, 2021.

[2] P. Shekhar, B. Csathó, T. Schenk, C. Roberts, and A. K. Patra. Alps: A unified framework for modeling time series of land ice changes. *IEEE Transactions on Geoscience and Remote Sensing*, pages 1–16, 2020.
